# A note on Weyl’s equidistribution theorem

**DOI:** 10.1007/s00605-025-02057-2

**Published:** 2025-02-14

**Authors:** Yuval Yifrach

**Affiliations:** University of Zurich, Zurich, Switzerland

**Keywords:** Equidistribution, Distribution modulo one, Weyl’s theorem, Lattice points, Multivariable polynomials, Homogeneous functions, Haar measure, Weak convergence, 11K36, 11K38, 37A17, 11B37

## Abstract

H. Weyl proved in Weyl (Eins Math Ann 77(3):313–352, 1916) that integer evaluations of polynomials are equidistributed mod 1 whenever at least one of the non-free coefficients (namely a coefficient of a monomial of degree at least 1) is irrational. We use Weyl’s result to prove a higher dimensional analogue of this fact. Namely, we prove that evaluations of polynomials on lattice points are equidistributed mod 1 whenever at least one of the non-free coefficients is irrational. This result improves the main result of Arhipov et al. (Mat Zametki 25(1):3–14, 157, 1979). We prove this analogue as a corollary of a theorem that guarantees equidistribution of grid evaluations mod 1 for all functions which satisfy some restraints on their derivatives. Another corollary we prove is that for $p\in (1,\infty )$ the $\ell ^p$ norms of integer vectors are equidistributed mod 1.

## Introduction

The following definition lies in the heart of all the results in this paper.

### Definition 1.1

Let $n\in \mathbb {N}$, let $\Gamma \subset \mathbb {R}^n$ a discrete set and let $F:D\subset \mathbb R^n\rightarrow \mathbb R$. We say that $F(\Gamma )\bmod 1$ is equidistributed if the sequence of discrete counting measures $\sigma ^D_R$ of $\Gamma$-points in $B_R(0)\cap D$ satisfies:

1.1 $$\begin{aligned} \lim _{R\rightarrow \infty }(F\bmod 1)_*\sigma ^D_R=\lambda \end{aligned}$$

in the weak sense, where $B_R(0)$ denotes the Euclidean ball of radius *R* around the origin in $\mathbb R^n$, $F\bmod 1:D\rightarrow \mathbb R/\mathbb Z$ is the composition of *F* with the quotient map, $(F\bmod 1)_*\sigma ^D_R$ denotes the push-forward measure, and $\lambda$ is the Haar probability measure on $S^1$, the unit circle.

H. Weyl proves in [2] that for any one variable polynomial *f* with at least one non-free irrational coefficient (namely a coefficient of a monomial of degree at least 1), $f(\mathbb {Z})\bmod 1$ is equidistributed. This result has various proofs (e.g. Weyl’s original proof in [2] and Furstenberg’s dynamical proof in [5, p. 116]) and generalisations (e.g. [3] for when instead of polynomials one considers a richer family of functions), including some results for joint distribution of several functions (see [3]). The main result of [4] shows that for any multivariable polynomial $F:\mathbb {R}^n\rightarrow \mathbb {R}$ with coefficients that satisfy certain Diophantine approximation conditions, $F(\mathbb {Z}^n)\bmod 1$ is equidistributed. A more natural (and in fact ’word to word’) extension of Weyl’s result is the following theorem:

### Theorem 1.2

Let $n\in \mathbb {N}$ and let $F:\mathbb {R}^n\rightarrow \mathbb {R}$, be a polynomial given by $F(x)=\sum _{\ell }\alpha _{\ell } x^{\ell }$ where $\ell =(\ell _1,\dots ,\ell _n)$ is a multi-index. Assume that there exists $\ell$ such that $|\ell |>0$ and $\alpha _{\ell }\notin \mathbb {Q}$, then $F(\mathbb {Z}^n)\bmod 1$ is equidistributed.

The method we use to prove Theorem 1.2 is general and is applicable for the proof of the following theorem as well:

### Theorem 1.3

Let $n\in \mathbb {N}$ and let $M\in \operatorname {GL}_n(\mathbb {R})$. Let $S\subset S^{n-1}$ be spherical cap inside the unit Euclidean sphere in $\mathbb {R}^n$ (namely, a subset of the sphere bounded by the intersection of a hyperplane with the sphere) and let $f:\mathbb {R}_+\cdot S\rightarrow \mathbb {R}$ denotes a smooth homogeneous (namely $f(sx)=sf(x)$ for all $x\in \mathbb {R}_+\cdot S$ and $s\in \mathbb {R}_+$) function where for a subset $I\subset \mathbb {R}_+$, $I\cdot S:=\{ta:t\in I,a\in S\}$. Assume there exists $v\in M\mathbb {Z}^n$ such that:

$$\begin{aligned} \left( \frac{\partial f}{\partial v}\bmod 1\right) _*m_S\ll \lambda \end{aligned}$$

where $\lambda$ is the Haar probability measure on $S^1$ and $m_S$ is the restriction of the $S^{n-1}$ area measure to *S*. Then for every $u\in \mathbb {R}^n$, $f(M\mathbb {Z}^n+u)\bmod 1$ is equidistributed.

### Remark 1.4

The most general statement that can be proved along the lines of Theorems 1.2,1.3 is given below as Theorem 2.4. For sake of keeping the introduction concise, we restricted this introduction to the two theorems above. We point out that Theorem 2.4 is heavily used in [6] to prove the main theorem.

Already as weaker form of Theorem 2.4, Theorem 1.3 can be used to prove the following corollary:

### Corollary 1.5

Let $n\in \mathbb {N}$ and $1<p<\infty$. Then $\left\Vert \cdot \right\Vert _{p}(\mathbb {Z}^n)\bmod 1$ is equidistributed, where $\left\Vert \cdot \right\Vert _p$ is the $\ell _p$-norm on $\mathbb {R}^n$.

## Proof of the Main Theorem

We need the following definition.

### Definition 2.1

*(Directional derivatives)* Let $U\subset \mathbb {R}^n$ be open, let $x\in U$ and let $f:U\rightarrow \mathbb {R}$ be smooth. For any $v\in \mathbb {R}^n$ and for small enough $\epsilon >0$ (depending on *v*, *x*, *U*), we define the function $f_v:(-\epsilon ,\epsilon )\rightarrow \mathbb {R}$ by $f_v(t)=f(x+tv)$. Given $d\in \mathbb {N}$, the *d*’th derivative of *f* in direction *v* is defined as $\frac{\partial ^d f}{\partial v^d}:=f_v^{(d)}(0)$.

We define a family of measures on the unit circle $S^1$. This family is used to state the main result, namely Theorem 2.4. The importance of this family will become clear with the statement of Theorem 2.4.

### Definition 2.2

Let $n\in \mathbb {N}$ and $M\in \operatorname {GL}_n(\mathbb {R})$. Let $\mu$ be a probability measure on $S^1$, $m_{S^{n-1}}$ be the Haar probability measure on $S^{n-1}$ and let $S\subset S^{n-1}$ be a spherical cap. Let $f:S\cdot \mathbb {R}_+\rightarrow \mathbb {R}$ be a smooth function. We denote $m_S$ to be the restriction of $m_{S^{n-1}}$ to *S*. We say that *f* is $M-S$-related to $\mu$ if there exists $d\in \mathbb {N}$ and $v\in M\mathbb {Z}^n$ such that:

2.1 $$\begin{aligned}  &   \frac{\partial ^{d} f}{\partial v^{d}}\text { is degree-} 0\text { -homogeneous (i.e. invariant under multiplication by positive scalars)},\nonumber \\ \end{aligned}$$

2.2 $$\begin{aligned}  &   \qquad \qquad \quad \qquad \qquad \left( \frac{\partial ^{d} f}{\partial v^{d}}\bmod 1\right) _*m_{S}=\mu \end{aligned}$$

and

2.3 $$\begin{aligned} \frac{\partial ^{d+1} f}{\partial v^{d+1}}(x)\rightarrow 0\hbox { as}\ \left\Vert x\right\Vert _2\rightarrow \infty . \end{aligned}$$

When $S=S^{n-1},M=I$ and the above holds we say simply that *f* is related to $\mu$.

### Remark 2.3

The discussion in this section can be carried in a slightly more general context. Namely, we can replace $S^1$ with the *k*-dimensional torus for $k\in \mathbb {N}$. The arguments and definitions will be identical to the case $k=1$ up to the appropriate modifications in the notation. For simplicity of this note, we chose not to treat this case.

In the following theorem we state equidistribution results for functions related (in the sense of Definition 2.2) to two families of measures.

### Theorem 2.4

Let $n\in \mathbb {N}$, $M\in \operatorname {GL}_n(\mathbb {R})$, $S\subset S^{n-1}$ a spherical cap and recall that $\lambda$ is the Haar probability measure on $S^1$. Let $\mu$ denote a probability measure which is either absolutely continuous w.r.t. $\lambda$ or supported on one irrational point in $S^1$ (namely a point of the form $e^{2\pi i\alpha }$ for $\alpha \in \mathbb {R}\setminus \mathbb {Q}$). Then for any smooth $f:S\cdot \mathbb {R}_+\rightarrow \mathbb {R}$ which is $S-M$-related to $\mu$ and for any $u\in \mathbb {R}^n$, $f(M\mathbb {Z}^n+u)\bmod 1$ is equidistributed.

We united the two claims of the above theorem because their proofs are conceptually the same. As first step towards proving Theorem 2.4, we need to define discrepancy and introduce some notation:

### Definition 2.5

Given $N,d\in \mathbb {N}$, $\epsilon >0$ and a sequence $\{x_n\}_{n\in \mathbb {N}}\subset \mathbb {R}$, we define:

- The discrepancy of $\{x_n\}_n$ after *N* steps by:
  $$\begin{aligned} D_N(\{x_n\}_n)=\sup _{I=[a,b]\subset [0,1]}|\mu _N(I)-(b-a)| \end{aligned}$$
  where $\mu _N$ is the uniform measure on the points $\{x_n\bmod 1\}_{n=1}^N\subset [0,1]$;
- The set:
  $$\begin{aligned}  &   A^d(N,\epsilon )=\{a\in [0,1]:D_l\left( \{[an^d+P(n)]\bmod 1\}_{n\in \mathbb {N}}\right) >\epsilon , \\  &   \text { for some polynomial }P\text { of degree at most } d-1 \text { and every }l=1,\dots ,N\}. \end{aligned}$$

As a second step we need the following lemma:

### Lemma 2.6

For any $d\in \mathbb {N}$ and $\epsilon >0$ the following assertions hold:

- For every $$N\in \mathbb {N}$$, $$A^d(N+1,\epsilon )\subset A^d(N,\epsilon )$$;
- $$\bigcap _{N\ge 1}A^d(N,\epsilon )\subset \mathbb {Q}\cap [0,1]$$, therefore $$\lambda (A^d(N,\epsilon ))\rightarrow 0$$ as $$N\rightarrow \infty $$.

### Proof

The first assertion is immediate upon noticing that enlargement of *N* adds conditions to the definition of $A^d(N,\epsilon )$. The second follows from the celebrated Erdős-Turan inequality since the bound on the exponential sum of $(an^d+P(n))_{n\in \mathbb {N}}$ for irrational *a* is uniform in *P* (where $\deg (P)<n$) by the remark following Equation (9) in [2]. $\square$

Now we are ready to prove Theorem 2.4.

### Proof of Theorem 2.4

Before we begin we remark that the notation *a* will usually denote an element of $S^1$, which is sometimes be thought of as [0, 1) under the identification $[0,1)\ni t\mapsto e^{2\pi it}\in S^1$. Let $\mu$ be a measure on $S^1$ satisfying the conditions of the theorem, $S\subset S^{n-1}$ be a spherical cap and let $f:S\cdot \mathbb {R}_+\rightarrow \mathbb {R}$ be a smooth function which is $S-M$-related to $\mu$ with some $d\in \mathbb {N}$. Let $\epsilon ,\delta >0$, $N_0\in \mathbb {N}$ to be determined. Define for any $T>0$:

$$\begin{aligned} B_{T,\epsilon }:= &   \{p\in (M\mathbb {Z}^n+u)\cap [0,T]S: \frac{\partial ^d f}{\partial v^d}(p)\bmod 1\notin A^d(N_0,\epsilon )\} \\= &   \{p\in (M\mathbb {Z}^n+u)\cap [0,T]S: \frac{\partial ^d f}{\partial v^d}(p/\left\Vert p\right\Vert _2)\bmod 1\notin A^d(N_0,\epsilon )\} \end{aligned}$$

where the equality holds by (2.1) (recall Definition 2.5). Recall Definition 1.1 and denote for all $T>0$:

$$\begin{aligned} \sigma _T^S=\sigma _T^{S\cdot \mathbb {R}_+} \end{aligned}$$

to be the counting measure of $M\mathbb {Z}^n+u$ in $S\cdot \mathbb {R}_+$. We claim that in both cases: where $\mu$ is supported on an irrational point and where $\mu \ll \lambda$, we can make sure that for some $\epsilon _k\rightarrow 0$, large enough $N_0=N_0(\epsilon _k,\delta )$ and $T>T_0(\epsilon _k,\delta )$:

2.4 $$\begin{aligned} \sigma _T^S(B_{T,\epsilon _k}^c)\le C_2\delta \end{aligned}$$

for some fixed $C_2>0$ depending only on *M* (recall that $B_{T,\epsilon }$ depends on $N_0$). Indeed, suppose that $\mu$ is supported on an irrational point, call it $\alpha \in S^1$. In this case, the irrationality of $\alpha$ and Lemma 2.6(2) imply that for all $\epsilon >0$:

2.5 $$\begin{aligned} \text {for all large enough }N_0, \alpha \notin A^d(N_0,\epsilon ). \end{aligned}$$

Otherwise, suppose that $\mu \ll \lambda$. Then by Lemma 2.6 (2) and since $\mu \ll \lambda$, for all $\epsilon >0$:

2.6 $$\begin{aligned} \text { for all large enough }N_0, \mu (A^d(N_0,\epsilon ))<\delta . \end{aligned}$$

Now let $\mu$ satisfy either one of the conditions of Theorem 2.4. Denote $\pi :S\cdot \mathbb {R}_+\setminus \{0\}\rightarrow S$ to be the projection onto S, namely $x\mapsto x/\left\Vert x\right\Vert _2$. It is well known (see [7]^1^) that there exists $0<C_1<C_2$ depending on *M* and a probability measure ${\tilde{m}}_{S}$ on *S* such that

- $$\pi _*\sigma ^S_T\rightarrow {\tilde{m}}_S$$ as $$T\rightarrow \infty $$;
- $$C_1m_S\le {\tilde{m}}_S\le C_2m_{S}$$.

Therefore for all $\epsilon >0$:

2.7 $$\begin{aligned} \sigma _T^S(B_{T,\epsilon }^c)= &   \pi _*\sigma _T^S(\{p\in S:\frac{\partial ^d f}{\partial v^d}(p)\bmod 1\in A^d(N_0,\epsilon )\})\xrightarrow {T\rightarrow \infty } {\tilde{m}}_S(\pi (B_{\infty ,\epsilon }^c)) \nonumber \\\le &   C_2m_S(\pi (B_{\infty ,\epsilon }^c))=\left( \frac{\partial ^d f}{\partial v^d}\bmod 1\right) _*m_S(A^d(N_0,\epsilon ))\nonumber \\\le &   {\left\{ \begin{array}{ll} 0,&  \text {when }\mu =\delta _{\alpha }\text { by equations }(2.5),(2.2); \\ C_2\delta ,&  \text {when }\mu \ll \lambda \text { by equations }(2.6),(2.2), \end{array}\right. } \end{aligned}$$

by the definition of $B_{T,\epsilon }$, the remark on the weak convergence of $\pi _*\sigma _T^S$. Thus for all *T* large enough equation (2.4) holds. To use the weak convergence in (2.7), we still need to verify that:

2.8 $$\begin{aligned} m_S\left( \partial \left[ \left( \frac{\partial ^d f}{\partial v^d}\bmod 1\right) ^{-1}A^d(N_0,\epsilon )\right] \right) =0 \end{aligned}$$

for an appropriate family of $\epsilon$’s that converge to 0. Since ${\tilde{m}}_S\le C_2m_S$, this will show the same for ${\tilde{m}}_S$ which is sufficient to apply the weak convergence in (2.7).

For any natural *k* and polynomial *P* of degree at most $d-1$, define a function $G_k(P,\cdot ):[0,1]\rightarrow \mathbb {R}_+$ by:

$$\begin{aligned} [0,1]\ni a\mapsto D_k\left( \left\{ ([an^d+P(n)]\bmod 1\right\} _{n\in \mathbb {N}}\right) \in \mathbb {R}_+. \end{aligned}$$

Denote $\mu _{a}$ to be the uniform measure on $\{[a1^d+P(1)]1,\dots ,[ak^d+P(k)]\bmod 1\}\subset S^1$ for any $a\in [0,1)$. Fix $a\in [0,1)$ and let $[0,1)\ni a_n\rightarrow a$. Let $I\subset [0,1)$ be an interval and let *m* be large enough such that $\mu _{a_n}(I)\le \mu _{a}(I+[-1/m,1/m])$ for every $n\ge m$. Such *m* can be found since we only consider finitely many points. Therefore:

$$\begin{aligned} \sup _{I\subset S^1}|\lambda (I)-\mu _{a}(I)|\ge \sup _{I\subset S^1}|\lambda (I)-\mu _{a_n}(I)|-\frac{2}{m}. \end{aligned}$$

Since this inequality is symmetric (we can replace $\theta ,\theta _n$), we get continuity of $G_k(P,\cdot )$. By similar arguments it is readily seen that $G_k$ is also continuous in the coefficients of *P*. Note that by definition of $D_k$ (see Definition 2.5) the function $G_k(P,\cdot )$ only depends on the values of the coefficients of *P* modulo 1 so in fact:

$$\begin{aligned} G_k(a):=\sup _{{\overline{b}}=(b_1,\dots ,b_d)\in [0,1]^d}G_k(P_{{\overline{b}}},a)=\sup _{P \hbox { Polynomial of degree}\ \le d-1}G_k(P,a) \end{aligned}$$

where for ${\overline{b}}=(b_1,\dots , b_d)$ we denote $P_{{\overline{b}}}(t)=b_1t^{d-1}+\dots +b_d$. Therefore $G_k(\cdot )$ is continuous as the supremum on the first parameter of $\tilde{G_k}:[0,1]^d\times S^1\rightarrow \mathbb {R}_+$, where $\tilde{G_k}({\overline{b}},a):=G_k(P_{{\overline{b}}},a)$ (it holds since the domain of $\tilde{G_k}$ is compact). Note that by definition of $A^d(N,\epsilon )$ and of $G_k$, $A^d(N_0,\epsilon )=\{a\in [0,1):G_k(a)>\epsilon \text { for every }k=1,\dots ,N_0\}$ therefore by continuity of $G_k(\cdot )$:

2.9 $$\begin{aligned} \partial A^d(N_0,\epsilon )=\{a\in [0,1):G_k(a)=\epsilon \text { for some }k=1,\dots ,N_0\}. \end{aligned}$$

Note that for every *k*, the set $C_k=\{t\in \mathbb {R}_+:\mu (\{G_k=t\})>0\}$ must be countable (otherwise we would find uncountably many disjoint subsets of $S^1$ with positive $\mu$ measure which contradicts the assumption on $\mu$), so $C=\bigcup _{k\ge 0}C_k$ is countable as well. In particular, we can take $\epsilon _k\rightarrow 0$ such that $\epsilon _k\notin C$ for every *k*. Note that by (2.9), for every *N*, $\partial A(N,\epsilon )\subset \bigcup _{M\le N}\{G_M=\epsilon \}$ and if $\epsilon \notin C$ we get in particular that $\mu (\partial A(N,\epsilon ))=0$. Recall the following general fact: for any continuous function $g:X\rightarrow Y$ between metric spaces, and a subset $A\subset Y$ it holds that $\partial (g^{-1}A)\subset g^{-1}(\partial A)$. Therefore by (2.2):

$$\begin{aligned}  &   m_S\left( \partial \left[ \left( \frac{\partial ^d f}{\partial v^d}\bmod 1\right) ^{-1}A(N,\epsilon _k)\right] \right) \le m_S\left( \left( \frac{\partial ^d f}{\partial v^d}\bmod 1\right) ^{-1}\partial A(N,\epsilon _k)\right) \\  &   \quad =\mu (\partial A(N,\epsilon _k))=0 \end{aligned}$$

so we justified the use of weak convergence in equation (2.7) with the sequence $(\epsilon _k)_k$.

For every $\epsilon >0$ and $p\in B_{\infty ,\epsilon }$, denote:

- $$I_p=\{p+kv:k=1,\dots ,N_p\}$$;
- $$f_p(t)=f(p+tv);t\in [0,N_p]$$;
- $$P_p(t)=\frac{\partial ^d f}{\partial v^d}(p)t^d+\dots +f(p)$$,

where $N_p$ is an integer in $[1,N_0]$ for which $G_{N_p}(\frac{\partial ^d f}{\partial v^d}(p))<\epsilon$ (which exists since $p\in B_{\infty ,\epsilon }$). By (2.3), for $T>T_0$ large enough $|\frac{\partial ^{d+1}f}{\partial v^{d+1}}(p)|<\epsilon /N_0^d$ when $p\in [T,\infty )S$. By Taylor’s Remainder theorem:

2.10 $$\begin{aligned} \left\Vert f_p-P_p\right\Vert _{\infty }\le C\epsilon \end{aligned}$$

for any $p\in [T,\infty )S$, after possibly modifying $T_0$ and for some absolute constant *C* (not depending on $N_0$). By (2.10) and Definition 2.5, for any $p\in B_{T,\epsilon }$ ($T>T_0$):

2.11 $$\begin{aligned} D_{N(p)}(\{f_p(k)\}_{k\in \mathbb {N}})\le C\epsilon \end{aligned}$$

for some absolute constant *C*. By equation (2.4) we may find for any $k\in \mathbb {N},\delta >0$ positive numbers $T>0,N_0\in \mathbb {N}$ large enough, and points $\{p_i^T\}_{i=1}^{N_T}\subset B_{T,\epsilon _k}$ for some $N_T\in \mathbb {N}$, such that:

2.12 $$\begin{aligned}  &   \frac{|\bigsqcup _{i=1}^{N_T}I_{p_i^T}|}{|[0,T]S\cap (M\mathbb {Z}^n+u)|}\ge (1-C_2\delta )+o_T(1); \end{aligned}$$

2.13 $$\begin{aligned}  &   I_{p_i^T}\subset [0,T]S\cap (M\mathbb {Z}^n+u). \end{aligned}$$

Note that the above two geometric properties follow from Equation (2.4) and the fact that $v\in M\mathbb {Z}^n$. Now by equations (2.11),(2.12),(2.13) we deduce that for any $k\in \mathbb {N}, I\subset S^1$ and *T* large enough:

$$\begin{aligned} |\sigma _T^S\left( (f\bmod 1)^{-1}I\right) -\lambda (I)|\le C_2\delta +(1-C_2\delta +o_T(1))C\epsilon _k \end{aligned}$$

which implies the claim of the theorem, by taking $T\rightarrow \infty$ and then taking $k\rightarrow \infty$, $\delta \rightarrow 0$ and using the fact that $\epsilon _k\rightarrow 0$. $\square$

### Remark 2.7

Although in Definition 2.2*S* is a subset of $S^{n-1}$, a larger family of sets can replace it in Theorem 2.4. For example, any open bounded subset *S* of a level surface of a homogenous function, if *S* is Jordan measurable in the level surface. Since the proof is identical we chose to treat the case of the sphere which is easier to visualise.

### Remark 2.8

In the proof, we required the following fact. Let $M\in \operatorname {GL}_n(\mathbb {R})$ and let $u\in \mathbb {R}^n$. For simplicity, we deal with the case $S=S^{n-1}$. The case of general *S* is similar. Denote for any $R>0$, by $\pi _*\sigma ^{M,u}_R$ the measure on $S^{n-1}$ coming from projecting the points $M\mathbb {Z}^n+u\cap B_R(0)$ to $S^{n-1}$ with the map $\pi$. Then $\pi _*\sigma _R\rightarrow {\tilde{m}}_{S^{n-1}}$ weakly as $R\rightarrow \infty$ where $C_1m_{S^{n-1}}\le {\tilde{m}}_{S^{n-1}}\le C_2m_{S^{n-1}}$. The reference [7] shows this claim for $M=I$ and $u=0$. We sketch a proof of how to deduce the general case from this. Let $A\subset S^{n-1}$ be open and Jordan measurable. We know from the case $M=I,u=0$ that $\pi _*\sigma ^{I,0}_R(\mathbb {R}_+\cdot M^{-1}A\cap S^{n-1})\rightarrow m_{S^{n-1}}(\mathbb {R}_+\cdot M^{-1}A\cap S^{n-1})$ as $R\rightarrow \infty$. Moreover, one readily checks that there exist $0<C_1<C_2$ depending on the operator norm of *M* such that $C_1 m_{S^{n-1}}(A)\le m_{S^{n-1}}(\mathbb {R}_+\cdot M^{-1}A\cap S^{n-1})\le C_2m_{S^{n-1}}(A)$. Define ${\tilde{m}}_{S^{n-1}}(A)=m_{S^{n-1}}(\mathbb {R}_+\cdot M^{-1}A\cap S^{n-1})$. Next, note that for any $u\in \mathbb {R}^n$ and $M\in \operatorname {GL}_n(\mathbb {R})$ there exists $C=C(M)>0$ such that $\pi _*\sigma _R^{M,u}(A)=\pi _*\sigma _{C(M)R}^{I,0}(\mathbb {R}_+\cdot M^{-1}A\cap S^{n-1})+o_R(1)$. Taking $R\rightarrow \infty$ shows $\pi _*\sigma _R^{M,u}(A)\rightarrow {\tilde{m}}_{S^{n-1}}(A)$ and we already know that ${\tilde{m}}_{S^{n-1}}$ satisfies $C_1m_{S^{n-1}}\le {\tilde{m}}_{S^{n-1}}\le C_2m_{S^{n-1}}$.

## Proof of Theorem 1.2 and Corollary 1.5

We start by proving Theorem 1.2. Our method is finding, for any polynomial *F* as in Theorem 1.2, $v\in \mathbb {Z}^{n}$ such that *F* and *v* satisfy equations (2.1)–(2.3) for $d=\operatorname {deg}(F)$ and a measure $\mu$ on $S^1$ which is supported on an irrational point. In the language of Definition 2.2, we will find a Dirac measure $\mu$ supported on an irrational point such that *F* is related to $\mu$. This will prove Theorem 1.2 using Theorem 2.4. First, we prove the existence of such *v* under an extra assumption:

### Lemma 3.1

Let $m,n,d\in \mathbb {N}$ and let $F:\mathbb {R}^n\rightarrow \mathbb {R}$ be $F(x)=\sum _{\ell }\alpha _{\ell }x^{\ell }$ where $\ell =(\ell _1,\dots ,\ell _n)$ is a multi-index and $d=\max \{|\ell |:\alpha _{\ell }\ne 0\}$ is the degree of *F*. Assume that there exist $\ell$ such that $|\ell |=d$ and $\alpha _{\ell }\notin \mathbb {Q}$. Then there exists $v\in \mathbb {Z}^n$ and an irrational point $\alpha _0\in (0,1)$ such that:

$$\begin{aligned} \frac{\partial ^{d} F}{\partial v^{d}}\equiv \alpha _0. \end{aligned}$$

In other words, *F* is related to $\delta _{\alpha _0}$.

### Proof

Assume for the sake of contradiction that:

3.1 $$\begin{aligned} \forall v\in \mathbb {Z}^n:\frac{\partial ^d F}{\partial v^d}\in \mathbb {Q}. \end{aligned}$$

Let $A_{n,d}=\{\ell :|\ell |=d\}$. For every $v=(m_1,\dots ,m_n)\in \mathbb {Z}^n$ let $u_v:A_{n,d}\rightarrow \mathbb {Z}$ be defined by:

3.2 $$\begin{aligned} u_v(\ell _1\dots ,\ell _n)=\prod _{i=1}^nm_i^{\ell _i} \end{aligned}$$

of course $\alpha ,u_v$ can be thought of as an element of $\mathbb {R}^{k=|A_{n,d}|}$. By direct calculation, Equation (3.1) implies that for every $v\in \mathbb {Z}^n$:

3.3 $$\begin{aligned} \sum _{\ell \in A_{n,d}}q_{\ell }\alpha _{\ell }u_v(\ell )\in \mathbb {Q}\end{aligned}$$

for some rational numbers $q_{\ell }$. By (3.2) there exist $v_1,\dots ,v_k\in \mathbb {Z}^n$ such that $u_{v_1},\dots ,u_{v_k}$ is a basis for $\mathbb {R}^k$. For example, we can take $v_i=(2^{i-1},\dots ,p_m^{i-1})$ for $i=1,\dots ,k$ where $p_{j}$ is the *j*’th prime. The vectors $u_{v_1},\dots ,u_{v_k}$ will form a basis for $\mathbb {R}^k$ since they are the columns of a Vandermonde matrix (this follows from (3.2)) and by the uniqueness of the prime decomposition, the entries of the vector $u_{v_2}$ are all different. This shows that the determinant will be non-zero. Therefore by the above equation every $\mathbb {Q}$-linear combination of elements from the set $\{\alpha _{\ell }:|\ell |=d\}$ is rational which contradicts the assumption. $\square$

Second, we prove Theorem 1.2:

### Proof of Theorem 1.2

Assume that the theorem holds for all polynomials of degree strictly less than *d*. If $\alpha _{\ell }$ is rational for every $|\ell |=d$, there exists $q\in \mathbb {Z}$ such that $q\alpha _{\ell }\in \mathbb {Z}$ for any $|\ell |=d$. Then the quantities $F(qv+r)\bmod 1$ for $v\in \mathbb {Z}^n$ and fixed $r\in \{0,\dots ,q-1\}^n$ coincide with values of polynomials of degree strictly smaller than *d* for which the conditions of the theorem hold. By induction we get that the values of *F* are equidistributed modulo 1. Therefore we may assume that $\alpha _{\ell }\notin \mathbb {Q}$ for some $|\ell |=d$. Lemma 3.1 shows that under the assumptions of Theorem 2.4, *F* is related to $\mu :=\delta _{\alpha _0}$ for some irrational $\alpha _0\in (0,1)$. By Theorem 2.4, $F(\mathbb {Z}^n)\bmod 1$ is equidistributed, as desired. $\square$

We conclude this note with a proof of Corollary 1.5. Similarly to the proof of Theorem 1.2, we do it by finding a direction $v\in \mathbb {Z}^n$ and a measure $\mu$ such that the norm function together with *v* and $\mu$ satisfy (2.1)–(2.3). Then we prove that $\mu$ satisfies the conditions of Theorem 2.4 and deduce uniform distribution.

### Proof of Corollary 1.5

Fix $p\in (1,\infty )$ and let $F:\mathbb {R}^n\rightarrow \mathbb {R}_+$ be defined by $x\mapsto \left\Vert x\right\Vert _{p}=\left( \sum _{i=1}^n|x_i|^p\right) ^{1/p}$. Since for any $i=1,\dots ,n$ *F* is invariant under the map $e_i\mapsto -e_i,e_j\mapsto e_j;\forall j\ne i$ it is sufficient to prove that $F\mid _{\mathbb {R}_+^n}\bmod 1(\mathbb {Z}^n)$ is equidistributed. Denote $v=e_j$ for some $j=1,\dots ,n$ (arbitrarily chosen). Note that *F* is homogeneous of degree 1 therefore $\frac{\partial }{\partial x_j}F$,$\frac{\partial ^2}{\partial x_j^2}F$ are homogeneous of degree $0,-1$ respectively and so, *F* satisfies Equations (2.1),(2.3) for $d=1$. It remains to check that (2.2) holds. Indeed, for any $(x_1\dots ,x_n)\in \mathbb {R}^n_+$:

3.4 $$\begin{aligned} \frac{\partial }{\partial x_j}F(x_1,\ldots ,x_n)=x_j^{p-1}\left( \sum _{i=1}^nx_i^p\right) ^{\frac{1}{p}-1} \end{aligned}$$

and denoting $S_p=\{x\in \mathbb {R}_+^n:\left\Vert x\right\Vert _p=1\}$, the function $G(x_1,\dots ,x_n):=\frac{\partial }{\partial x_j}F\mid _{S_p} (x_1,\dots ,x_n)=x_j^{p-1}$ satisfies $(G\bmod 1)_*\mu _{S_p}\ll \lambda$ where $\mu _{S_p}$ is the surface measure on $S_p$, since the degree of the Jacobian of *F* is 1 at every point. By Theorem 2.4, $F\mid _{\mathbb {R}_+^n}(\mathbb {Z}^n)\bmod 1$ is indeed equidistributed. $\square$

## Footnotes

1 This reference deals with the case $M=I$ and $u=0$ and yields ${\tilde{m}}_S=m_S$. However, the general case of $M\in \operatorname {GL}_n(\mathbb {R})$ and $u\in \mathbb {R}^n$ follows from this case. See Remark 2.8 for more details.
